# Education and Empowering Special Forces to Eradicate Secret Defectors: Immune System-Based Treatment Approaches for Mature T- and NK-Cell Malignancies

**DOI:** 10.3390/cancers15092532

**Published:** 2023-04-28

**Authors:** Till Braun, Alexandra Schrader

**Affiliations:** 1 Department I of Internal Medicine, Center for Integrated Oncology, Aachen-Bonn-Cologne-Duesseldorf, Excellence Cluster for Cellular Stress Response and Aging-Associated Diseases, Center for Molecular Medicine Cologne, University of Cologne, 50937 Cologne, Germany; till.braun@uk-koeln.de; 2 Lymphoma Immuno Biology Team, Equipe Labellisée LIGUE 2023, Centre International de Recherche en Infectiologie, INSERM U1111-CNRS UMR5308, Faculté de Médecine Lyon-Sud, Hospices Civils de Lyon, Université Claude Bernard Lyon I-ENS de Lyon, 69921 Lyon, France

**Keywords:** T- and NK-cell leukemia/lymphoma, WHO classification 2022, immunotherapy, antibody-drug-conjugate, ADCC/CDC, CAR-T-cell therapy

## Abstract

**Simple Summary:**

Mature T- and NK-cell leukemia/lymphoma (MTCL/L) are a heterogeneous group of rare, mostly poor prognostic neoplastic entities. In the past, immunotherapeutic approaches have evolved, leading to prolonged survival of many patients with solid tumors or B-cell malignancies. As there are now many new immune system-based approaches for MTCL/L on the horizon, we aimed to summarize the distinct immunotherapeutic approaches. The specificity of an immunotherapeutic approach in MTCL/L is that the immune system must attack a cell from its own ranks. Within this review, we will present how the special forces of the immune systems must be educated and empowered to eradicate the secret defectors. Hereby, we will focus on monoclonal as well as bispecific antibodies, immune-checkpoint blockades, and CAR T cell therapies.

**Abstract:**

Mature T- and NK-cell leukemia/lymphoma (MTCL/L) constitute a heterogeneous group of, currently, 30 distinct neoplastic entities that are overall rare, and all present with a challenging molecular markup. Thus, so far, the use of first-line cancer treatment modalities, including chemotherapies, achieve only limited clinical responses associated with discouraging prognoses. Recently, cancer immunotherapy has evolved rapidly, allowing us to help patients with, e.g., solid tumors and also relapsed/refractory B-cell malignancies to achieve durable clinical responses. In this review, we systematically unveiled the distinct immunotherapeutic approaches available, emphasizing the special impediments faced when trying to employ immune system defense mechanisms to target ‘one of their own—gone mad’. We summarized the preclinical and clinical efforts made to employ the various platforms of cancer immunotherapies including antibody-drug conjugates, monoclonal as well as bispecific antibodies, immune-checkpoint blockades, and CAR T cell therapies. We emphasized the challenges to, but also the goals of, what needs to be done to achieve similar successes as seen for B-cell entities.

## 1. Introduction

Immune evasion is a central hallmark of malignant transformation, allowing the uncontrolled growth and proliferation of cancer cells [1]. In recent years, immunotherapies, which enable the immune system to recognize and effectively eradicate cancer cells, have become a fundamental part of cancer treatment regimens [2]. The first observations of immune reaction-induced anti-cancer effects go back to the late 19th century when the orthopedic surgeon William B. Coley observed regressions of osteosarcomas upon operation-induced inflammations [3]. After that, Coley successfully treated a variety of tumors by inducing inflammatory reactions through injections of a wild mixture of different bacteria (including, e.g., Streptococcus pyogenes). However, employing the ‘Coley’s toxin’ came with obviously dangerous adverse effects and was, based on the limited understanding of involved anti-cancer mechanisms, not further followed up on. During the journey of exploring molecular dependencies involved in cancer immunoediting, physicians tried to empower cancer patients’ immune systems by inducing enhanced T cell activation and proliferation with only moderate success. Those strategies included, for example, the use of high-dose IL2 treatment for patients with melanoma or renal cell carcinoma [4] (this strategy has been further improved by developing more defined genetically engineered IL2 ‘muteins’ affecting specifically CD8^+^ cytotoxic T cells) or direct T cell co-activation using an anti-CD28 super-agonistic antibody. The latter was evaluated in a Phase I clinical trial, resulting in severe clinical illness and the hospitalization of all six healthy volunteers due to a cytokine storm-induced systemic inflammatory response [5].

**Figure 1:**
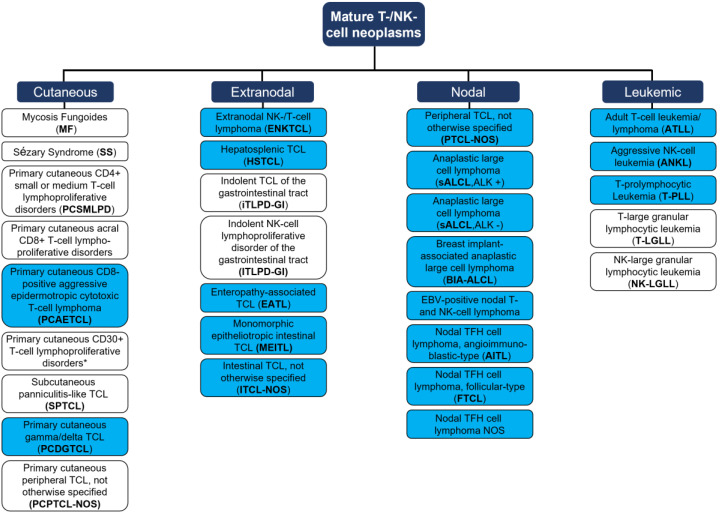
Mature T cell and NK-cell neoplasms, according to the WHO classification of hematolymphoid tumors (2022, 5th edition). Blue = aggressive entity; white = indolent entity. One distinct group of ‘EBV positive T- and NK-cell lymphoid proliferations and lymphomas of childhood’ are not presented as no data on immunotherapies for those entities are available. * Two differential clinical entities: (i) Lymphomatoid papulosis; (ii) Primary cutaneous anaplastic large cell lymphoma. TCL = T cell lymphoma. TFH = T-follicular helper. Adapted from Alaggio et al. [9].

Since then, advanced research has focused on the development of carefully fine-tuned immunomodulatory interventions accounting for the difficile balance of anti-cancer effects and autoimmunity [6]. Those immunotherapies largely rely on the reactivation of adaptive immune responses that were influenced and thus silenced by the tumor cell, or that are evaded by the tumor cells via disguising mechanisms [7]. In the scope of this review, we questioned how to employ immunotherapies against mature T- and NK-cell leukemia/lymphoma (MTCL/L) that arise from exactly this population of defender cells belonging to the adaptive immune system. MTCL/Ls compromise a heterogeneous group of malignancies, characterized by the clonal expansion of post-thymic T cells [8]. According to the novel WHO classification of 2022, the group MTCL/Ls represents a growing field with, currently, 30 different entities [9] that can be grouped into primary cutaneous, nodal, extranodal, or leukemic diseases (Figure 1, please see Figure for abbreviations). The heterogeneity of T- and NK-cell malignancies is reflected by their varying tissue-specific distributions, as well as by differing T cell functionalities of neoplastic cells (e.g., active cytotoxic T cells vs. resting T helper cells) [10]. Differences further translate into prognostic perspectives [11] with, e.g., T-LGLL presenting as an indolent disease resulting in overall survival of around one decade [12], vs. most other peripheral T cell lymphoma (PTCL) and T-PLL presenting as highly aggressive malignancies [13].

First-line therapies for most MTCL/L are composed of intense chemotherapy regimens. These include cyclophosphamide, doxorubicin, vincristine, etoposide, and prednisone (CHO(E)P [14]) for most PTCL or dexamethasone, methotrexate, ifosfamide, L-Asparaginase, and etoposide ((m)SMILE [15]) for ENKTL, with discouraging responses for virtually all entities, rendering new treatment approaches urgently warranted [11]. Upon complete remission (CR) in response to first-line treatment and sufficient physical fitness, patients can subsequently undergo (autologous or allogeneic) stem cell transplantations (SCT). However, these curative approaches only reveal survival rates of 40–50% for most entities [16]. Notably, only three immune system-based therapies have ever achieved approval by drug administration agencies for the treatment of only a small subgroup of MTCL/L, with only two remaining FDA approved medications to date: the anti-CD30 antibody–drug conjugate Brentuximab vedotin (ALCL and CD30^+^ CTCL; first-line in 2018) [17]; the anti-CD52 antibody Alemtuzumab (originally for B-cell CLL in 2007, withdrawal from market for oncologic applications in 2012) [13,18], and the anti-CCR4-receptor antibody Mogamulizumab for SS and MF (second-line in 2018), as well as CCR4^+^ ATLL (in Japan 2018) [19,20]. In the case of relapsed/refractory (r/r) disease, only a few targeted substances are approved (e.g., the histone deacetylase (HDAC) inhibitor Belinostat), showing unsatisfactory responses [21].

In this review, we discussed the established and currently-tested preclinical and clinical efforts to overcome the specific pitfalls of anti T- and NK-cell immunotherapies such as the selection of a T cell neoplasm-selective target, since targeting a pan T cell antigen is associated with T cell aplasia and, therefore, higher morbidity and mortality [22]. We systematically summarized the various platforms of cancer immunotherapies in the treatment of MTCL/L, including monoclonal antibodies (i) employed as antibody-drug conjugates mediating a targeted delivery of chemotherapeutics, (ii) as targeted inducers of complement-/cell-mediated cytotoxicity, (iii) as immune-checkpoint inhibitors targeting PD-1/PD-L1 and CTLA4 to reactivate halted T cell response, or (iv) engineered to contain dual binding moieties able to recruit activated NK-cells to target cells. Furthermore, we included cell-mediated treatment strategies involving allogeneic T cell transplants as well as CAR-expressing T and NK cells. We highlighted the deviances but also illustrated potential strategies to achieve similar successes as seen for the therapy of B-cell entities. To our knowledge, here we present the first review summarizing immunotherapeutic strategies for the treatment of MTCL/L in their entirety.

## 2. Monoclonal Antibodies in the Treatment of Mature T- and NK-Cell Malignancies

As an immunotherapeutic approach, mAbs have already been established in the clinical routine. To date, they are applied in first-line therapy for some MTCL/L entities, exemplarily in the treatment of sALCL or T-PLL [23,24]. In the following, we summarize preclinical as well as clinical efforts of mAb-based therapy of MTCL/L, depending on the mechanism of action of the respective mABs; as (i) vehicles to transport highly effective cytotoxins (so-called antibody-drug conjugates), as (ii) inducers of complement- and cell-mediated cytotoxicity, and as (iii) immune checkpoint inhibitors (see Table 1 for overview).

### 2.1. Antibody-Drug Conjugates

In general, chemotherapies are limited by their severe systemic cytotoxic side effects. To improve the specificity of cytostatic drugs, antibody-drug conjugates (ADC)—a combination of immunotherapy with chemotherapy—can be employed [25,26]. Mediating a targeted effect towards epitope-carrying malignant cells, a highly selective mAB is linked to a potent cytotoxic agent that can induce target cell death upon internalization. Following this strategy, higher drug concentrations at tumor sites and limited systemic exposure to drugs are achieved. Thus, lower overall doses are needed and more potent drugs can be used, nevertheless reducing systemic toxicity and allowing for better tolerability of the drug.

A prerequisite for successful mAB-mediated therapies is a carefully selected antigen with restricted or no expression in healthy cells. Target antigens are classified either as tumor-specific antigens (TSAs) or as tumor-associated antigens (TAAs), depending on the expression of the respective antigen on the physiological T cell compartment. To achieve successful tumor eradication without severe side effects, the choice of targeted antigens is of the utmost importance [27]. This is especially the case in T cell malignancies, as targeting surface molecules non-specifically expressed by the entirety of T cells (pan-T-cell antigens) induces severe T cell aplasia due to ‘on-target off-tumor effects’, leading to higher morbidity and mortality [22]. Within the last decades, the cell-surface molecule CD30, primarily exploited for the targeted treatment of Hodgkin lymphoma (HL), also emerged as a target for MTCL/L. Despite sALCL, showing CD30 positivity in virtually all cases, other MTCL/L also exhibit CD30 expression in a subgroup of cases and/or even in a subpopulation of lymphoma cells. Usually, CD30 expression is assessed using immunohistochemical analyses of tissue specimens. However, cutoffs for positivity are only defined for sALCL (‘positive’ if ≥75% of cells are positive) with no clearly defined percentages for other MTCL/L [28]. Considering cases with >1% of CD30^+^ cells as positive, the following entities reveal considerable CD30 expression: PTCL-NOS (32–64% of cases), AITL (46–80% of cases), ATLLs (0–55%), ENKTL (46–80% of cases), and EATL (50–100% of cases). Among CTCL, CD30 expression has been reported in 59% of patients with non-transformed MF and 100% of patients with transformed MF [29,30].

Following up on the clinical success of Brentuximab vedotin (BV) in the treatment of HL, a large-scale Phase III clinical trial (ECHELON-2) was initiated to evaluate the efficacy of CD30-targeted therapy, in combination with CHP (Cyclophosphamide, Doxorubicin, Prednisone), in 452 patients with CD30 positive PTCL (≥10% of cells by local review) including ALK-positive sALCL, ALK-negative sALCL, PTCL-NOS, AITL, ATLL, EATL, and HSTL [17]. BV is an ADC consisting of the cytotoxic agent monomethyl auristatin E (MMAE) linked to an anti-CD30 humanized IgG1 monoclonal antibody. Upon CD30 binding, antibody-target complexes are internalized and MMAE induces microtubule disruption leading to cell cycle arrest and apoptosis [28]. Horwitz and colleagues showed that treatment with BV-CHP reduced the risk of death by 34% compared to CHOP treatment in the entirety of patients included (‘intention to treat’ cohort comprising cases of all entities). BV-CHP was improving overall survival also, specifically for non-sALCL histologic subtypes (PTCL-NOS, and AITL). Based on this clinical trial, BV-CHP was approved as a first-line treatment for sALCL by the EMA and, additionally, for CD30^+^ PTCL by the FDA.

Evaluating the clinical efficacy of BV in primary cutaneous MTCL/L, Prince and colleagues conducted an open-label, randomized, Phase III, multicenter trial enrolling 131 patients with CD30^+^ MF or primary cutaneous ALCL (≥10% CD30-positive malignant cells or lymphoid infiltrate by central review) who had been previously treated (ALCANZA [31]). The study compared the proportion of patients achieving an objective global response (ORR = Objective Global Response Rate) lasting at least 4 months in groups of intravenous (i.v.) BV single treatment vs. physician’s choice (oral methotrexate or bexarotene). This comparison strongly favored BV treatment with 56.3% (36 of 64 patients) vs. 12.5% (eight of 64 patients) of patients achieving the primary endpoint. Following this, BV was approved as a single agent in second-line therapy for CD30^+^ CTCL.

Furthermore, current clinal studies are evaluating potential combinations of BV with other therapeutic strategies for the treatment of CD30^+^ MTCL/L: (i) drugs showing single agent activity such as Lenalidomide (NCT03409432), (ii) targeted therapies such as Romidepsin (NCT02616965), (iii) plain mAbs inducing complement-/cell-mediated cytotoxicity such as Mogamulizumab (NCT05414500), and (iv) immune checkpoint-inhibitors such as anti-PD-1 antibodies tislelizumab/pembrolizumab (NCT05316246/NCT04795869).

Despite those promising results, a drawback of BV therapy remains the standardized detection of CD30 expression as the only biomarker applicable for the prediction of clinical responses. There is no consensus among pathologists regarding the critical percentage of CD30^+^ cells necessary for sufficient BV effects and the discrimination of CD30^+^ lymphoma cells from CD30^+^ activated cells can be challenging for some lymphoma entities (e.g., AITL) [32]. Thus, current clinical trials evaluate the efficacy of BV, especially in MTCL/L cases with <10% CD30+ cells (NCT02588651; NCT04569032).

With respect to MTCL/L entities, where the choice of immunomodulatory treatments might be especially complicated, ADCs, such as BV especially, stand out, as their mode of action does not require the patient’s adaptive immune response for treatment success. In this case, immune system-derived tools (mAbs) are solely employed for drug delivery–leaving the cancer-defending capacities of patients’ immune cells alienated.

### 2.2. Antibodies as Agents Conveying Complement-/Cell-Mediated Cytotoxicity

Despite the strategy of using antibodies as vehicles to transport chemotherapeutics to target cells, plain mAbs themselves can serve as effective therapeutic agents by marking malignant cells for complement-dependent cytotoxicity (CDC) and antibody-dependent cell-mediated cytotoxicity/phagocytosis (ADCC/ADCP). As an example, Rituximab, an unconjugated mAb directed against CD20 [33], constitutes a central component of most treatment regimens against B-cell malignancies [34]

Complement-dependent cytotoxicity (CDC), a mechanism generally in charge of defending against intruding pathogens, represents an integral component of the innate immune system, mediated via activation of a proteolytic cascade involving more than 30 different proteins [35]. Complement system initiation can be achieved, among other signals, by antibody-immune complex recognition. The most important subsequent effects lead to (i) direct lysis of targeted surfaces by the formation of the membrane attack complex (MAC), (ii) production of proinflammatory anaphylatoxins leading to immune system priming, and (iii) opsonization of pathogens and activation of phagocytic cells (macrophages or neutrophils). ADCC and ADCP depend, as the name suggests, on mAb binding to the target cell (-antigens). Effector cells of the innate immune system, classically NK-cells, macrophages, or neutrophils, express Fc receptors (e.g., FcγRIIIb/CD16) on their surface, which, upon binding to the mAb’s Fc region, cross-link and activate. Subsequently, several intracellular signaling cascades are induced, resulting in (i) the release of cytotoxic granules and thus target cell killing via, e.g., the perforin/granzyme cell death pathway, or (ii) antibody-dependent phagocytosis of the target cell [33,36].

#### 2.2.1. Targeting CD52 by Alemtuzumab

The cell surface glycoprotein CD52 is particularly highly expressed on several T cell lymphoma entities including AITL, HSTL, T-PLL, and CTLC cells and other hematologic malignancies such as B-cell chronic lymphocytic leukemia [37,38]. As CD52 is also present in healthy mature lymphocytes, monocytes, and dendritic cells (but not on hematopoietic stem cells), CD52 presents one example of a protein structure conveying dangerous ‘on-target-off tumor effects’, when targeted [39]. Clinical data from several Phase I/II studies for the use of Alemtuzumab, a humanized IgG1 antibody directed against CD52, were promising for some PTCL entities such as CTCL, including MF, SS, T-PLL, and T-LGLL [18,40,41,42,43]. However, recently, a randomized open-label Phase III clinical trial was conducted, investigating the combination of Alemtuzumab with CHOP vs. CHOP alone in a patient cohort of 116 elderly PTCL patients (including diagnoses of PTCL-NOS, AILT, FTCL, MEIL, ALCL, ENKTL [44]). The study by Wulf and colleagues concluded that Alemtuzumab failed to improve the outcome; although Alemtuzumab addition to the CHOP regime showed positive effects on CR rates and primary progression, those were counterbalanced by dose-limiting complications of high infection rates, especially from viral (CMV and EBV) and fungal agents (also previously described [45]). Additionally, the study observed treatment-associated secondary malignancies (diffuse large B-cell lymphomas), which were likely to arise from EBV-infected B-cells. Those complications most probably occurred due to immune system failures based on the depletion of healthy CD52-carrying T cells (on-target off-tumor effects). Overall, Alemtuzumab seems to constitute an effective therapeutic option, specifically for patients with highly aggressive leukemic disease. Nevertheless, its use is limited due to high rates of serious infectious complications, based on the T cell ubiquitous expression, and thus lacking tumor cell specificity, of CD52. Furthermore, Alemtuzumab was withdrawn for hematologic and oncologic indications from US and European markets in 2012 due to strategic reasons. Since then, the antibody has been reintroduced as a treatment for multiple sclerosis, making it available for compassionate use in CD52 high-expressing MTCL/L entities such as T-PLL, T-LGL, MF, and SS.

#### 2.2.2. Targeting CCR4 by Mogamulizumab

Those severe immune-depletion-induced side effects might be overcome by targeting a surface antigen that is only expressed by a T cell subpopulation (and the targeted lymphoma/leukemia cells). In the physiologic state, the chemokine receptor CCR4 is present on the surface of T helper type 2 (Th2) cells, CD4^+^ cells, circulating cutaneous lymphocyte-associated antigen (CLA)-positive skin-homing T cells, and regulatory T cells (Tregs) [46,47]. Furthermore, high expression levels of CCR4 have been detected on the surface of lymphoma/leukemia cells in different stages of MF and SS [48], with CCR4-mediated signaling being important for MF/SS’s pathophysiology. CCR4 activation facilitates the trafficking of leukemic cells into the skin and thus promotes disease progression by microenvironmental effects. Furthermore, ATCL cells show high levels of CCR4 expression, being associated with cutaneous manifestations and poor prognosis [49]. To target CCR4 expression in MF/SS [50,51] and ATCL [52], the fully humanized, anti-CCR4 antibody Mogamulizumab (IgG1) was developed. Mogamulizumab binds to the N-terminal domain of CCR4 with high affinity and promotes ADCC, not interfering with CCR4’s signaling and thus pro-tumorigenic function [53,54]. For an improved ADCC via high-affinity binding to the FcγRIII on effector cells, the Fc part was selectively defucosylated (glycoengineered) [55]. Mogamulizumab’s mode of action seems to go beyond mere tumor cell killing: as Tregs play an important role in the pathophysiology of cutaneous T cell lymphoma, a part of the therapeutic effect is also attributed to the eradication of CCR4^+^ Tregs in the skin. As the first glycoengineered antibody on the market, Mogamulizumab has emerged as an effective therapeutic agent: In a randomized Phase III clinical trial, Kim and colleagues evaluated the efficacy of Mogamulizumab compared to the HDAC inhibitor Vorinostat for second-line treatment of MF or SS [19]. First analyses of this study revealed an improved progression-free survival (PFS) for patients treated with Mogamulizumab compared to Vorinostat therapy (median PFS 7.7 vs. 3.1 months). Notably, an association of Mogamulizumab treatment with the occurrence of Stevens–Johnson Syndrome, an immunological intolerance reaction, which leads to detachment of the skin, was described. However, Mogamulizumab was approved by the FDA and the EMA for second-line treatment of MF and SS in 2018 [19] and for CCR4^+^ ATLL, r/r CCR4^+^ PTCL, and r/r CTCL in combination with chemotherapy regimens in Japan [56]. Overall mogamulizumab seems to be a valuable new therapeutic option for r/r CTCL, being generally tolerable and effective. However, drug-induced skin toxicities can be problematic, especially as they can be hard to discriminate from disease progression events.

#### 2.2.3. Targeting CD38 by Daratumumab and Isatuximab

In the search for novel, specific target structures, the anti-CD38 mAbs Daratumumab and Isatuximab (both IgG1) are currently under investigation for the treatment of a subset of T cell lymphoma/leukemia entities [57,58]. In physiological conditions, CD38 surface expression is restricted to thymocytes, activated T cells, NK cells, and plasma cells. Resting T cells do not express CD38. Interestingly, CD38 is expressed on cells of certain mature T cell malignancies including subgroups of AITL and PTCL-NOS, as well as most ENKTL cases [59]. For ENKTL CD38 has been identified as a prognostic maker [60]. In a global approach, CD38 surface expression was assessed for 51 unique MTCL/L specimens using flow cytometry data that has been previously acquired for diagnostic purposes [61]. CD38 was expressed to some extent in *n* = 49 of *n* = 51 cases including PTCL-NOS, AITL, FTCL, sALCL, T-LGLL, T-PLL, CTCL, ATLL, HSTCL, and MEITL [61]. Additional hematological malignancies expressing high levels of CD38 include multiple myeloma, chronic lymphocytic leukemia, mantle cell lymphoma, acute lymphoblastic leukemia, acute myeloid leukemia, and plasma cell leukemia. CD38 is a type II transmembrane glycoprotein, acting both as an ectoenzyme (NADase/ADPR cyclase) and as a receptor involved in cell signal transduction [62,63]. Upon binding to targeted cells, anti-CD38 directed antibodies, in addition to mediating CDC, ADCC/ADCP, can induce apoptosis and can modulate CD38’s enzymatic activity. Daratumumab was first approved in 2015 for patients with multiple myeloma (MM). By now it is applied for the treatment of r/r and newly diagnosed MM showing excellent improvements in clinical efficacy (mostly in combination strategies) [64,65].

Following up on a case report from 2014 [66], Daratumumab monotherapy was tested for patients with r/r ENKTL in a multicenter Phase II study [58]. Huang and colleagues observed an ORR of 25% with a median progression-free survival (PFS) of only 53 days, concluding that disregarding this limited efficacy of Daratumumab for ENKTL therapy, future approaches should evaluate combinations of anti-CD38 treatment with other agents previously used for ENKTL treatment. In line with this, a combination of an anti-CD38 antibody (Isatuximab) with the immune-checkpoint inhibitor Cemiplimab (anti PD-1 antibody) was recently evaluated for efficacy in PTCL in a Phase II clinical trial (NCT04763616). This study is based on the hypothesis that blocking PD-L1 on PTCL cells might restore healthy T cell mediated immune effector functions and may thus generate an additive or even synergistic immune-modulatory effect with CDC/ADCC/ADCP inducing Isatixumab. An interim analysis published at the beginning of 2023 showed a complete or partial response in only 9.1% of 11 included PTCL patients [57]. With these results, Carlo-Stella and colleagues concluded that, for PTCL patients, interim efficacy analysis results did not meet prespecified criteria to continue enrolment and that no synergistic effect could be observed. For CTCL, promising *in vitro* and *in vivo* analyses have recently shown CD38 surface expression on primary neoplastic cells from MF and SS patients. Xenograft models of CTCL cell lines showed responsiveness towards anti-CD38 antibody treatment [61,67].

#### 2.2.4. Targeting CD70 by Cusatuzumab

An additional surface molecule exploitable for lymphoma cell targeting might be CD70, which is physiologically expressed on activated T and B cells and mature dendritic cells in a transient fashion. CD70 expression has been documented in a variety of cancers, including PTCL and CTCL [68,69]. Functionally, CD70 is a member of the Tumor Necrosis Factor (TNF) superfamily of receptors which can be activated through CD27, thereby inducing pro-tumorigenic effects. Furthermore, CD70 activation via CD27 serves as a costimulatory signal, regulating immune responses [70], and Treg activation via the CD70-CD27 signaling axis has been proposed as one mechanism of tumor-immune-escape [71]. The human anti-CD70 defucosylated mAb Cusatuzumab (ARGX-110, IgG1) is designed to (i) block CD70 mediated signaling and thereby inhibit tumor cell growth, to (ii) induce tumor cell killing via ADCC, and to (iii) block CD70-CD27 mediated activation of Tregs, leading to reactivation of patients’ immune system (checkpoint blockade [72]). A Phase I/II clinical trial evaluating Cusatuzumab for the treatment of advanced CD70 positive (≥10% of tumor cells showing CD70 expression) CTCL in 27 patients demonstrated fair to modest clinical activity (ORR = 23%), with 9 patients achieving SD and one case showing a CR for 3 years (NCT01813539 [73]). Overall, the authors conclude that, despite the observed moderate efficacy of Cusatuzumab, the favorable safety profile allows testing it in a multidrug approach in combination with currently used treatment regimens. In addition, clinical activities of antibody-drug conjugates (ADCs) targeting CD70 in B-cell NHL have recently been evaluated [74], with preclinical data on xenograft models of patient-derived CTCL cells showing promising *in vivo* efficacy of an anti-CD70 ADC [69].

#### 2.2.5. Targeting KIR3DL2 by Lacutamab

Interestingly, it has been noticed for several years that malignant T cells can phenotypically change/transdifferentiate towards cells of other immune lineages, including NK cells [75,76]. Among phenotypic changes, lymphoma cells alter their surface marker composition, exemplarily, expression of the killer immunoglobulin-like receptors (KIR) family members can be induced. KIR3DL2 is physiologically expressed on cytotoxic lymphocytes such as NK cells, which mediate their effector functions through binding to their cognate MHC class I ligands. Regarding T cells, KIR3DL2 expression is restricted to less than a few percent of CD8 and CD4 T cells. However, previous studies demonstrated KIRDL2 expression on primary cutaneous T cell lymphoma as well as ATLL cells.

To target lymphoma cells, the anti-KIR3DL2 mAb Lacutamab (IPH4102, IgG1), a humanized monoclonal anti-KIR3DL2 antibody was developed [77]. Preclinical data revealed promising results for Lacutamab, efficiently enabling autologous NK-cells to eliminate KIR3DL2^+^ primary ATLL cells in *ex vivo* assays [78]. In addition to ATLL, a Phase I trial for CTCL evaluated the safety and activity of Lacutamab in a cohort of 44 r/r CTCL patients (NCT02593046) [79]. Authors reported no dose-limiting toxicity with the most common adverse events being low-grade peripheral edema (27%) and fatigue (20%), and lymphopenia as the most common grade 3 or worse adverse event (7%). A confirmed global OR was achieved in 36% of cases, with nearly all of them being SS cases. Bagot and colleagues conclude that Lacutamab is safe and shows encouraging clinical activity, particularly in SS. Furthermore, the U.S. Library of Medicine lists 3 distinct, ongoing, Phase II trials evaluating Lacalutamab in PTCL: (i) The TELLOMAK trial evaluates Lacutamab as a single agent for r/r MF and SS (NCT03902184). Furthermore, (ii) another Phase II study evaluates Lacutamab (in combination with Gemcitabine and Oxaliplatine) vs. standard of care (Gemcitabine and Oxaliplatine alone) in patients with r/r KIR3DL2^+^ (≥1% of tumor cells), nodal as well as extranodal PTCL (NCT04984837). An additional trial tests the safety and efficacy of Lacutamab in r/r KIR3DL2^+^ (≥1% based on central evaluation by IHC) PTCL patients disregarding the PTCL subtype (NCT05321147). Especially results from trials evaluating Lacutamab-chemotherapy combinations might eventually help to improve also first-line treatments of MTCL/L and thus allowing advanced patient outcomes.

#### 2.2.6. Targeting ICOS by MEDI-570

T-cell activity and survival are, in addition to antigen recognition by the T cell receptor, tightly regulated via co-stimulatory and co-inhibitory signals (e.g., PD-1/PD-L1; CTLA4, CD28/CD80, CD86; ICOS/ICOS-L). Exploiting re-activation of healthy T cells to eliminate malignant MTCL/Ls via targeting those T cell co-inhibitors will be discussed in the following chapter. However, despite the development of activating and inhibitory mAbs targeting ICOS, also mAbs intended for CDC/ADCC induction upon mAb-target cell binding have been developed. The co-stimulatory receptor ICOS (CD278) is physiologically expressed at a low level on activated resting memory T cells, and at high levels on follicular T helper cells and regulatory T cells [80]. Essentially, ICOS is known to be highly expressed on PTCLs derived from follicular T-helper cells (such as AITL, FTCL, and a proportion of PTCL-NOS) [81,82]. ICOS-L is ubiquitously expressed on somatic cells. The co-stimulatory signal mediated by ICOS activation plays an important role in memory and effector T cell development and immunosuppressive signals mediated by Tregs. To target ICOS, MEDI-570, a human afucosylated, IgG1 mAb was developed [83]. The first Phase I clinical trial was conducted in patients with systemic lupus erythematosus (NCT01127321), based on the observation that ICOS-expressing T cells were increased in these patients. Recently, the first results from a Phase I trial for dose finding and evaluation of preliminary efficacy of MEDI-570 in r/r T-follicular helper phenotype PTCL and AITL were published (NCT02520791) [84]. The study included heavily pretreated patients with a median of 3 prior lines of therapies. Overall, Chavez and colleagues conclude that efficacy analysis of MEDI-570, especially for AITL patients (ORR of 30%, with 2 patients showing CR) showed promising activity and a manageable safety profile, suggesting further confirmatory studies, especially in AITL. Furthermore, the preclinical efficacy of an ADC derived from mAbs targeting ICOS was shown recently *in vitro* and *in vivo* using CTCL cell lines and xenograft models of primary cells isolated from CTCL patients [85]. In addition to targeting ICOS-expressing MTCL/L cells, the MEDI-570 mediated therapeutic effect may, at leads in part, be attributed to the killing of ICOS-positive regulatory T cells and thus boosting of an anti-tumoral immune response.

#### 2.2.7. Empowering the Innate Immune System by Blocking the ‘Do Not Eat Me’ Signal

One major limitation of mAb therapy, utilizing cell-mediated cytotoxicity, is the inability of the innate immune system to recognize the secret defector. As one strategy of immune escape, the malignant cells upregulate CD47, the so-called ‘do not eat me’ signal. Abolishing the binding of CD47 to its receptor signal-regulatory protein α (SIRPα) on macrophages and dendritic cells by an anti-CD47 antibody can result in phagocytosis reactivation [86,87]. A currently ongoing Phase I/II clinical trial (NCT04541017) evaluates the efficacy of combining Mogamulizumab with the anti-CD47 antibody Magrolimab (Hu5F9-G4) for the treatment of r/r MF/SS. Apart from this, Magrolimab is currently tested for the treatment of other hematologic malignancies e.g., r/r B-cell lymphoma [88], acute myeloid leukemia [89,90], and follicular lymphoma [88]. Additionally, several other anti-CD47 and anti-SIRPα directed antibodies, as well as inhibitory fusion proteins are currently in clinical development [86], offering a promising perspective allowing effective MTCL/L therapies through reinforcing recognition and killing of MTCL/L cells through the innate immune system.

MTCL/L development, and thus expansion of one malignant T cell clone, is often accompanied by a disturbed T cell homeostasis, leading to loss or severe impairment of healthy functional T cells. As immunotherapies employing mAbs, mostly designed to induce CDC and ADCC/ADCP mediated lymphoma cell killing, rely on effector functions of the innate immune system, the strategies listed above might be the most convenient approaches to improve MTCL/L therapies.

### 2.3. Antibodies as Immune-Checkpoint Inhibitors

To enable recognition and killing of malignant cells through the adaptive immune system (T-cell activation by antigen-presenting cells and subsequent killing of target cells via cytotoxic T cells [91]), reactivation of remaining healthy T cells can be achieved via immune-checkpoint inhibitors. Two prerequisites for this strategy are (i) the sufficient availability of residual healthy T cells and (ii) the independence of lymphoma cell growth from checkpoint-mediated signaling, a particular pitfall when targeting T cell lymphomas using T cell reactivators. Two important immune-checkpoint signals that regulate T cell activity, and that can be targeted for anti-cancer therapies, are PD-1–PDL-1 and CTLA-4–CD80/CD86 interactions [21,92].

#### 2.3.1. Disrupting CTLA4-Mediated Pro-Survival Signaling Applying Ipilimumab

In principle, checkpoint-mediated signaling can be understood as an immune reaction-‘off-switch’ that keeps T cell reactivity in check to prevent exaggerated immune responses and auto-immune effects. In the physiological setting, full T cell activation is achieved only upon antigen recognition by the TCR through binding of MHC I/MHC II molecules in combination with co-stimulatory signals mediated via the CD28 receptor (on T cells) and its ligands CD80/CD86 (on APCs) [93]. One way to inhibit T cell activation is conveyed via the CD28 homolog CTLA4, whose expression is induced on activated T cells. CTLA4 has a higher binding affinity for CD80 and CD86 and can thus abolish CD28-mediated co-stimulatory signals, thereby inhibiting T cell activation [94,95]. Furthermore, Treg-mediated immune tolerance is centrally involving CTLA4. Tregs use CTLA4 on their surface to block co-stimulatory signals for other T cells and to purposefully trans-endocytose CD80 and CD86 present on APC’s surfaces via trogocytosis [96,97,98,99]. In solid cancers such as melanoma, renal cell carcinoma, hepatocellular carcinoma, non-small cell lung cancer, and colorectal cancer, the anti-CTLA4 mAb Ipilimumab has been approved by the FDA either as mono- or as combination therapy with an anti-PD-L1 antibody (Nivolumab/Pembrolizumab) in first- and second line treatments [100]. Regarding hematologic malignancies, Ipilimumab was recently evaluated for safety and effectivity in patients with r/r Hodgkin Lymphoma (ORR 76%), B-cell NHL (ORR 24%), and FL (ORR 4–20%) [101,102,103,104,105], with several other clinical trials still ongoing. For MTCL/L no systematic clinical data on Ipilimumab are available and no studies are currently ongoing. However, based on a case report showing a good clinical response in a patient with MF AND melanoma (Ipilimumab was used for melanoma treatment [106]) it seems worthwhile to try. Furthermore, with respect to tumor cell pathophysiology, targeting CTLA4 might be especially interesting in cases that have acquired CTLA4 mediated pro (!)-survival signaling. Genetic analyses revealed that a notable percentage of PTCLs (58% of AITLs, 23% of PTCL-NOS, and 29% of ENKTLs) harbor *CTLA4-CD28* fusion genes, encoding for the extracellular part of CTLA4 (high affinity to CD80/CD86 ligands) combined with the intracellular part of CD28 (pro-tumorigenic signaling) that show pro-proliferative effects upon ectopic expression in cell line systems *in vitro* [107] A case report presenting data on the clinical response of an SS patient harboring the respective genetic lesion showed that those *CTLA4-CD28* fusions are in principle targetable using Ipilimumab [108].

#### 2.3.2. Preventing Immune Escape Pathways by Targeting PD-1/PD-L1

In addition to CTLA-4, peripheral T cell activity is regulated via PD-1–PD-L1 interactions. The co-inhibitory PD-1 receptor (CD279) is primarily expressed on activated T cells. Physiologically the ligand PD-L1 (CD274) is constitutively present on cells of various tissues including activated T and B lymphocytes, dendritic cells, monocytes, mesenchymal stem cells (MSCs), bone marrow (BM)-derived mast cells [109,110]. Tumor cells have evolved to hijack this immune-inhibitory pathway by overexpressing PD-L1 molecules leading to a pathologic inhibition of T cells encountering tumor cells and thus to immune system escape [111]. In solid cancers, targeting PD-1/PD-L1 T cell inhibitory signals via mAbs [e.g., Nivolumab (anti PD-1), Pembrolizumab (anti PD-1), Cemiplimab (anti PD-1), Avelumab (anti PD-L1), Durvalumab (anti PD-L1), Atezolizumab (anti PD-L1)] for immune-system reactivation has revolutionized the success of oncogenic therapies. In 2018, James Allison and Tasuku Honjo were awarded with the Nobel Prize in Physiology or Medicine for their contributions toward an improved understanding of the function of immune checkpoints in cancer [112]. By now, we know that PD-1/PD-L1 targeting results in overall increased numbers of T cells, increased cytotoxic functions and cytokine production, and, eventually, tumor cell lysis [92].

For MTCL/L surface expression of PD-1 and PD-L1 was detected on lymphoma cells of various entities, however with differing prevalence: One study reports PD-1-expression in 93% of AITL cases [113], while another series evaluating 168 PTCL cases showed positivity in only 62% of AITL [114]. With a cutoff of 10% positive cells in IHC this study found PD-1 expression also in 40% of PTCL-NOS, and 13% of ALK^−^ ALCL cases. ALK^+^ ALCL cases were found to exhibit low or no PD-1 expression [114]. Furthermore, Kim and colleagues reported PD-1 and PD-L1 positive tumor cells in 63% of AITL and 59% of PTCL-NOS cases (cutoff value of 5%.) [115]. For PD-L1 another study found 99% of PTCLs to be positive (>1% positive cells). The positivity was 56% if a cutoff of ≥50% was used [116]. Using the ≥50% cutoff, authors find 79% ENKTL, 71% ALK^+^ ALCL, 39% ALK^−^ ALCL, and 36% of PTCL-NOS cases positive for PD-L1. Overall, studies have shown that MTCL/L cells frequently express PD-1/PD-L1 molecules (and that respective expression levels can correlate with prognostic perspectives [117,118]). Interestingly, for ENKTL, PD-1 expression was rarely or not detected (0–2% of cases) while PD-L1 expression could be found in 56–80% of cases.

So far, a series of clinical trials have been conducted to evaluate immune-checkpoint inhibitors as mono- and combination therapies for the treatment of MTCL/L [21]. Here, we would like to summarize the overall results considering the specific ‘T-cell anti T cell lymphoma’ effects and report the most recent findings.

In 2019 a Phase II study evaluated the anti-PD-1 mAb Nivolumab in 12 patients with r/r AITL, PTCL-NOS, and ALK^−^ ALCL. The ORR was 33% (with two CRs, two PRs, and a median DOR of 3.6 months). Despite those positive effects, the treatment also induced a significant disease progression within one treatment cycle in four cases (‘hyperprogression’). Due to the moderate rate of positive effects and the high number of hyperprogression events, the study was discontinued [119]. In 2022 an interim analysis of another Phase II trial evaluating Nivolumab showed comparable results in a patient cohort of 12 cases including AITL, PTCL-NOS, ALK^-^ ALCL, EATL, and HSTCL [120]. The authors observed an ORR of 33% (two CR, two PR, and a median DOR of 3.6 months). Also in this study, 4 patients experienced therapy-induced hyperprogression and the study was halted. One additional study on the effects of the anti-PD-1 mAb Pembrolizumab in a cohort of 13 PTCL cases (including PTCL-NOS, FTL, and MF patients) was stopped early after interim analysis due to low efficacies of anti-PD-1 treatments. One patient experienced therapy-associated progression [121]. However, clinical studies evaluating Pembrolizumab in cohorts including dominantly ENKTL cases showed more promising results: For one study including 7 patients with r/r ENKTL an ORR of 100% was achieved (CR in 5 cases) [122], another study observed an ORR of 57% in a cohort of 7 patients (CR in 2 and PR in 2 cases) [123], and a third study on 30 patients with r/r NHL (including 14 ENKTL cases) accomplished an ORR of 44% (CR in 5 cases, PR in 1 case) [124]. Successes in ENKTL patient cohorts were furthermore followed up on in a Chinese Phase II trial evaluating the efficacy of the anti-PD-1 mAb Sintilimab in 28 r/r ENKTL cases. Investigators observed an ORR of 75% with CR and PR rates of 21 and 54%, respectively. A 2-year OS rate of 78.6% was observed [125]. The so-far largest trial evaluated the anti-PD-1 mAb Geptanolimab in a cohort of 102 r/r PTCL cases including PTCL-NOS, ENKTL, ALK^−^ ALCL, ALK^+^ ALCL, and others [116]. Investigators found an ORR of 40% (14% CRs; 26% PRs) and a DOR of 11.4 months with Geptanolimab showing a favorable safety profile. Cases of hyperprogession were not reported.

So far, for clinical studies evaluating anti PD-L1 mAbs such as CS1001 [126], or Avelumab [127], data are available for r/r ENKTL patients, reporting ORRs of 44% and 38% respectively. Furthermore, the AVAIL-T trial evaluated Avelumab in a cohort of 34 PTCL cases including AITL, PTCL-NOS, ENKTL, ALCL, and MF. The reported results showed a median OS of 8.9 months and a median PFS of 2.9 months (NCT03046953 [128]). No cases of hyperprogression were reported. However, authors conclude that with >50% of these patients not reaching the first evaluation point (15 days after completion of 3 cycles), and overall, only modest reductions in tumor size, interruption of PD1-PDL1 signaling does not appear effective in the setting of r/r PTCL.

Studies evaluating combinatorial approaches are ongoing and include combinations of Pembrolizumab with targeted therapies of HDAC inhibitors Romidepsin (NCT03278782) or Pralatrexate (NCT03598998). However, first results show similar obstacles comparable to single agent treatments such as only moderate ORRs and cases experiencing hyperprogression [129]. On the contrary, for ENKTL, combinations of PD-1–PD-L1 inhibitions with HDAC inhibition and chemotherapy regimens (chidamide, etoposide, and thalidomide) were tested in a small cohort of 3 cases showing an ORR of 100% [130]. Furthermore, the anti PD-L1 antibody Durvalumab is as well currently evaluated alone or in combination with lenalidomide for the treatment of r/r PTCL (NCT03011814), and in combination with pramipexole, romidepsin, and/or azacitidine (NCT03161223) for the treatment of T cell lymphoma [21]. Promising *in vitro* evaluations show a potential synergy of TTI-621-mediated CD47 inhibition (reactivation of macrophage-mediated phagocytosis) with anti-PD-L1 targeting antibodies [131]. However, additional analyses must be performed to exploit combinatorial T cell and macrophage reactivation approaches in MTCL/L.

Overall, clinical results show, so far, that PD-1/PD-L1 targeting seems to have only moderate success in MTCL/L from T cell origin but might serve as a promising strategy for ENKTL treatment with ORRs of up to 100%. It is reasonable to suspect that a moderate response rate and hyperprogession events in PTCL might be associated with immune-checkpoint signaling acting as a tumor suppressive pathway in certain lymphoma types [132,133]. This coherence further emphasizes the need for clinical studies which focus on biologically distinct subgroups and to, additionally, carefully evaluate biomarkers (e.g., PD-1 expression on lymphoma cells) in order to select patients eligible for anti-PD1-PD-L1/anti CTLA4 treatments.

## 3. Bispecific Antibodies

Despite blocking inhibitory signals that halt healthy T cells or macrophages through targeting immune checkpoints or ‘don’t eat me signals’ via mAbs, the recruitment of immune cells towards the malignant MTCL/L cells can as well be directly achieved using bispecific antibodies [134]. Bispecific antibodies constitute engineered molecules that comprise two antigen-recognition moieties. Most successful bispecific antibodies are so-called ‘bispecific T cell engagers’ (biTEs) that contain a target cell spotting antibody structure (e.g., anti-CD19) and an effector cell recruiting/activating antibody structure (e.g., anti-CD3). In this way, biTEs directly couple T cells and targeted neoplastic cells to form an immune synapse, resulting in T cell receptor (TCR) activation, the release of granzymes and perforin, and eventually target cell lysis [135]. Serving as role models for the development of biTEs in the treatment of immune-system derived neoplasia, Blinatumomab, an anti-CD19/anti-CD3 biTE, and Mosunetuzumab, an anti-CD20/anti-CD3 biTE, are already successfully applied in the clinical setting–being FDA and EMA approved for r/r B-precursor acute lymphoblastic leukemia and r/r Follicular Lymphoma respectively [136,137,138]. However, for MTCL/L, the application of bispecific antibodies is still restricted to experimental settings and focuses mostly on bispecific antibodies which recruit NK cells (and not T cells) towards the malignant T cell clone.

### 3.1. Pairing the Innate Immune System with CD30^+^ T cells

Also, for utilizing bispecific antibodies, the choice of an appropriate TAA/TSA is of utmost importance. Targeting CD30 in ALCL via BV has turned out to be successful even in first-line therapy [17]. With the development of the bispecific anti-CD30/anti-CD16A antibody AFM13, this tumor cell surface marker might be exploitable for another therapeutic strategy involving cells of the innate immune system [139]. The tetravalent bispecific antibody AFM13, with two binding sites for each CD30 and CD16A (FcγRIIIα), can recruit and activate CD16A^+^ NK cells to CD30-expressing tumor cells and can subsequently mediate tumor cell killing [140]. A Phase II clinical trial testing AFM13 for the treatment of patients with r/r HL showed only moderate treatment efficacies (16% of 25 included patients) [141]. However, one must take into account that those patients were heavily pretreated and did relapse even after autologous stem cell transplantation, BV, and anti-PD1 treatment. The U.S. National Library of Medicine currently lists 3 clinical trials (NCT03192202, NCT04074746, NCT04101331) evaluating the eligibility of AFM13 for r/r PTCL-NOS, MF, ALCL, and CTCL, however, only interim results have been published so far [142]. First results of the investigator-sponsored Phase I/II study in patients with r/r CD30^+^ CTCL (NCT03192202) were presented at the 2020 American Society of Hematology (ASH)-meeting: Data of 14 patients showed that AFM13 was well tolerated and showed single-agent therapeutic activity (ORR of 40%) [143]. According to Yago L. Nieto from the M.D. Anderson Cancer Center, the principal investigator of the NCT04074746 study, 19 heavily pretreated patients have been submitted to lymphodepletion followed by transfusion of AFM13–NK cell complexes. Due to the potential malfunctioning of autologous NK cells, investigators chose to co-transplant donor-derived cord-blood NK cells, which were preactivated and expanded *in vitro*, in form of an NK cell-AFM13 complex infusion into patients. Utilizing cord-blood-derived NK cells has been shown previously to be effective for lymphoma cell killing *in vitro* [140]. Among those patients, 10 cases exhibited CRs with an ORR of 90%. At 9 months, median PFS (52%) and OS (81%) rates were impressive. Overall, AFM13 seems to be a very promising immunotherapeutic approach for which preliminary clinical data show good tolerability and striking activity in patients, who have failed first-line therapies and have very limited treatment options.

### 3.2. Equipping T cell Engagers to Recognize Lymphoma/Leukemia-Specific TCR-Beta Chains

In order to eradicate MTCL/L cells, targeting pan T cell antigens is not feasible, as resulting global T cell aplasia will induce severe immune depression accompanied by dangerous infections as severe side effects [22]. Thus, the group around Vogelstein and colleagues has developed a strategy engineering a biTE that recognizes the TCR-beta chain subtype-specific for the malignant T cell clone [144] combined with an anti-CD3 recognizing part able to recruit and activate residual healthy T cells. In principle, each T cell expresses a unique TCR-beta chain generated from a repertoire of 30 distinct TCR β chain variable gene families (TRBV1 to TRBV30). Upon targeting of a single TRBV family member expressed by the malignant T cells (and a small subset of healthy T cells which accidentally used the same TCR-beta chain) killing of MTCL/L cells could be achieved, while the majority of healthy T cells (expressing one of the other 29 possible TRBV family members) remain untouched. The bispecific antibodies presented in this study can target T cells expressing TRBV5–5 (α-V5) or TRBV12 (α-V12). *In vitro* experiments using cell lines and primary human leukemic cells (T-ALL) together with T cells isolated from healthy donors showed a complete loss of the TRBV5–5^+^ and TRBV12^+^ expressing cells. Xenograft models transplanted with Jurkat cells (T-ALL cell line), together with primary human T cells as well showed successful eradication of transplanted malignant cells. However, the authors observed a phenomenon of bidirectional killing, by which the targeted malignant T cell is as well activated through the binding of the bispecific antibody and then subsequently attacks the healthy CD3-recruited effector T cell. This mechanism might be problematic for actual clinical applications. Overall, this approach holds promising new perspectives regarding the specification of appropriate TSAs but has to be tested further in additional preclinical experiments–maybe in combination with an anti-CD16 antibody part to achieve NK-cell recruitment.

**Table 1:** Synopsis of surface antigens targeted by therapeutic mAbs for the treatment of MTCL/L entities. Selected antibodies represent examples.

Mechanism	Target Antigen	Therapeutic Antibody	Clinical Trial/FDA or EMA Approval
drug delivery	CD30	Brentuximab-Vedotin	sALCL [17]. CTCL [31]
CDC, ADCC/ADCP, apoptosis induction	CCR4	Mogamulizumab	MF and SS [19] CCR4^+^ ATLL, CCR4^+^ PTCL, and CTCL [56].
	CD38	Daratumumab, Isatuximab	ENKTL [58].
	CD52	Alemtuzumab	MF, SS, T-PLL, and T-LGLL [18,40,41,42,43]. PTCL-NOS, AILT, FTCL, MEIL, sALCL, ENKTL [44]
	CD70	Cusatuzumab	CTCL [73]
	ICOS	MEDI-570	T-follicular helper phenotype PTCL and AITL [84].
	KIR3DL2	Lacutamab	CTCL [79].
NK-cell recruitment, macrophage reactivation	CD30 (& CD16)	AFM13	PTCL-NOS, MF, sALCL, and CTCL [142]; CD30^+^ CTCL [143]
	CD47	Magrolimab	MF/SS (NCT04541017)
T-cell reactivation	CTLA4	Ipilimumab	case report [108]
	PD-1/PD-L1	Nivolumab, Pembrolizumab, Cemiplimab, Sintilimab, Geptanolimab, CS1001, Avelumab, Durvalumab, Atezolizumab	AITL, PTCL-NOS, ALK^−^, sALCL [119] AITL, PTCL-NOS, ALK^−^, sALCL, EATL, and HSTCL [120] PTCL-NOS, FTL, MF [121] ENKTL [122,123,124,125,126,127]
T-cell recruitment	TRBV5-5/TRBV12	α-V5; α-V5	preclinical/experimental [144]

## 4. CAR T Cell Therapy in the Treatment of Mature T Cell Malignancies

Within the last decade, CAR T cell therapy has emerged and been successfully implemented in the therapy of relapsed/refractory lymphoma [145]. In line with the concept of arming special forces to eradicate secret defectors, this therapeutic approach uses mostly (autologous) T cells, isolated from the peripheral blood of the cancer patient. Until now, 5 distinct products have granted approval for the therapy of r/r B-cell leukemia/lymphoma and are currently working their way further and further toward first-line therapies: While axicabtagene ciloleucel (Yescarta^TM^), lisocabtagene maraleucel (Breyanzi^TM^), and tisagenlecleucel (Kymriah^TM^) have been approved for r/r diffuse large B-cell lymphoma [146,147], tisagenlecleucel has been additionally established for young adult patients with r/r acute lymphoblastic leukemia (ALL) [148]. Furthermore, idecabtagene vicleucel (Abecma^TM^) and ciltacabtagene autoleucel (Carvykti^TM^) have been established in the therapy of r/r multiple myeloma [149,150] and brexucabtagene autoleucel (Tecartus^TM^) in the therapy of r/r mantle cell lymphoma as well as r/r ALL [151]. In the following, we will summarize preclinical and clinical efforts of CAR T cell therapies in MTCL/L and will focus on challenges, that arise during the therapy of a T cell employing a T cell, and how to overcome them [152,153].

### 4.1. Principles of CAR T Cell Therapy

In general, isolated T cells are *in vitro* expanded and genetically modified to express a target-specific Chimeric Antigen Receptor (CAR). The CAR, a synthetic molecule that is surface-expressed, serves as a ‘weapon’ which allows effector cells, such as T cells or natural killer cells (NK cells), to focus their cytotoxicity specifically on those tumor cells that express the CAR-targeted antigen. CAR-expressing effector cells recognize target cell surface antigens using a single-chain variable fragment recognition domain and creating a non-classical immune synapse upon binding, which is crucial for their effective function. Subsequently, CAR effector cells exert their anti-target cell effects via several pathways, including the perforin and granzyme axis, the Fas and Fas ligand axis, and the release of cytokines, which sensitizes the tumor microenvironment [154].

Furthermore, a CAR consists of several critical components: An extracellular antigen-binding domain, a hinge domain, a transmembrane domain, a co-stimulatory domain (such as CD28), and an activation domain (from CD3ζ). Notably, the major histocompatibility complex (MHC) is not involved in antigen recognition via the non-classical immune synapse and thus in the activation of CAR-carrying T cells [155]. Therefore, CAR T cells are able to recognize antigens, that were not previously processed and presented by MHC II molecules on APCs. Consequently, any surface-expressed antigen that is primarily expressed on malignant cells (and can also be targeted using mAbs) may serve as an appropriate antigen target for CAR T cell therapy. Notably, there are two methods of introducing CAR transgenes into T cells: transiently (by mRNA electroporation) [156] or stably (by lentiviral or gammaretroviral transduction) [157]. While CAR T cell therapies have shown high response rates in relapsed/refractory B-cell lymphoma and have been, therefore, approved, no CAR T cell product has been granted in the treatment of mature T cell malignancies [153].

### 4.2. Challenges of T Cell-Targeted CAR T Cell Therapy

The nature of T cell directed CAR T cell therapy presents three fundamental challenges: (i) The expression of the CAR targeted antigen on the surface of CAR T cells themselves triggers a phenomenon recognized as fratricide, whereby CAR T cells engage in self-directed cytotoxicity and subsequently undergo apoptosis [158]. This event culminates in compromised CAR T cell persistence and ultimately diminishes the antitumor efficacy of the adoptive cell therapy. (ii) Again as an on-target off-tumor effect, CAR T cells can recognize and attack normal T cells that bear the same antigen as the tumor target, leading to T cell aplasia and, therefore, increasing the susceptibility of affected individuals to a spectrum of potentially lethal infections [153]. (iii) In addition, the production of autologous CAR T cells from patients afflicted with T cell malignancies poses a considerable obstacle as the isolation process inevitably captures both, malignant and healthy T cells. Consequently, the resulting CAR T-product population incorporates both normal and malignant T cells (product contamination) [152]. In the following, we will highlight recent investigations that aim to mitigate these caveats, thereby paving the way for improved CAR T-therapeutic strategies.

### 4.3. Selection of Target Antigens for CAR T Cell Therapy

In the realm of educating special forces, various strategies exist for identifying and selecting optimal targets. In the context of CAR-mediated immunotherapy, targeted antigens are classified as either TSAs or TAAs, such as the classification used for mAb-based therapy. Table 2 provides an overview of preclinical validations as well as clinical trials, studying CAR-based therapy in MTCL/L, presented for each targeted antigen.

The most commonly studied TAAs for CAR-based therapies of T cell malignancies are pan T cell antigens, such as CD3, CD5, and CD7 (Table 2). However, as these TAAs are also expressed on healthy T cell populations, CAR T cells must be equipped with additional armaments to prevent T cell depletion and fratricide (methods will be discussed in the following sections). Although TAA-targeting CAR T cells have shown promising results in naïve T cell entities, clinical data on their applicability in MTCL/L is limited to one Phase I trial (NCT03081910), which is currently recruiting, and, therefore, further trials are highly warranted.

In contrast to TAAs, antigens expressed only on a subset of T cells result in lower rates of T cell depletion and fratricide when used as CAR T cell antigens. Following up on clinical trials investigating CD4-specific mAbs [159], CD4-targeting CAR T cells have shown promising *in vitro* activity in primary PTCL samples and the ALCL cell line Karpas-299 [160]. Furthermore, the efficacy of CD4 CAR T cells was proven in a PTCL-like xenograft model. However, as the application of CD4 CAR T cells will again lead to a depletion of CD4^+^ T cells, patients treated with this construct might suffer from an AIDS-like syndrome. Therefore, further studies must implement techniques (as described below), to prevent T cell depletion of the healthy compartment, before moving further into clinical trials.

A further approach based on the selection of a T cell antigen with restricted expression builds up on the successful implementation of BV in PTCL, especially ALCL: CD30-targeted CAR T cells have shown strong preclinical efficacy and have been already implemented within clinical trials, mainly for patients with Hodgkin Lymphoma but also ALCL patients have been included. Up to now, data on efficacy is limited to small case series [161,162,163,164,165,166], and a systematic assessment is highly warranted.

In addition, the restrictive expression of either TRBC1 or TRBC2 on malignant clones was utilized as a target structure for the development of CAR T cells [167]. Notably, in a normal population of T cells, a 1:1 mixture of cells expressing either TRBC1 or TRBC2 is observed. Upon selecting a CAR directed against the TRBC1/2 subtype of the present malignant T cell clone, on-target-off-tumor effects of CAR T cells will deplete only half of the healthy T cell compartment, thus preventing T cell aplasia. Notably, the efficacy of TRBC-directed CAR T cells has been proven *in vitro* as well as *in vivo* (xenograft model utilizing the T-ALL such as Jurkat cell lines) [167], and a Phase I/II clinical trial in patients with TRBC1^+^ T-NHL is ongoing.

Additionally, the promising results of Mogamulizumab in MTCL/L have encouraged researchers to develop CAR T cells directed against CCR4: Allogeneic CCR4-redirected CAR T cells presented effective antitumor responses against CCR4-expressing patient-derived tumor cell lines *in vitro* [168]. Furthermore, these CAR T cells exhibited tumoricidal responses in a xenograft model of ATLL, highlighting the possible suitability of this antigen for the treatment of T cell malignancies. However, the expression of CCR4 on normal T cell subsets may lead to unexpected toxicities, such as Stevens–Johnson syndrome, which has been previously reported for Mogamulizumab and requires further analyses. Up to now, no clinical trial investigating CCR4-targeting CAR T cells has been initiated.

Further TSAs, which could be utilized for the engineering of CAR T cells, are the tetraspanin leukocyte-exclusive surface antigen CD37 [169], the extracellular matrix metalloproteinase inducer CD147, the cyclic ADP ribose hydrolase CD38, as well as the signaling lymphocytic activation molecule family member 7 (SLAMF7). Nonetheless, preclinical validations of these antigens are still in their infancy.

**Table 2:** Synopsis of preclinical and clinical trials evaluating CAR T-/NK-cell therapy in primary samples, cell lines, or *in vivo* models of MTCL/L, listed in dependence on the target antigen, Notably, the presented table focuses on MTCL/L, studies in naïve T cell entities have not been considered.

Target Antigen	*In Vitro* Validation	*In Vivo* Validation	Clinical Trial
CD3	[170]	none	none
CD5	[171,172]	none	NCT03081910 NCT05138458 *
CD7	[173]	[173]	NCT02742727 **
CD30	none	none	NCT01316146 [165] NCT02259556 [161] NCT02690545 [162,163,166] NCT02917083 [163] NCT02663297 [164]
CD4	[160,174]	[160,174]	NCT03829540 NCT04973527 NCT04712864 NCT04219319 NCT04162340
CD37	[169]	none	none
CCR4	[168]	[168]	none
TRBC1/2	[167]	none	NCT04828174 NCT03590574
CD147	none	none	NCT05013372

* clinical trial for CAR myeloid cell therapy ** clinical trial for CAR NK cell therapy.

### 4.4. Equipping CAR T Cells with Suicide Switches to Halt the Elimination of Target-Carrying T Cells and to Overcome T Cell Aplasia

As described above, the selection of the best suitable target is an essential step toward successful CAR T cell therapy in the treatment of MTCL/L. However, to prevent T cell aplasia even when using TAAs ubiquitously present on healthy T cells, a viable approach involves the incorporation of safety switches (also referred to as suicide switches) into CAR T cells. This allows for regulation of the adoptively transferred T cells following their introduction into patients. These safety switches have been first described for patients receiving allogeneic stem cell transplantation, to avoid graft-versus-host disease (GvHD) [175]. To date, various types of safety switches have been implemented in CAR-based approaches [155]:

- (i) Metabolic switches operate by inducing the conversion of a non-toxic compound into a toxic one, which results in the elimination of the cell carrying the suicide switch. The best-known approach is based on the transduction of a gene encoding for the herpes simplex virus thymidine kinase (HSV-TK) [176]. Upon the administration of ganciclovir, this virus static is subsequently phosphorylated to monophosphorylated and, then, triphosphorylated ganciclovir (TP) by the HSV-TK. TP is incorporated into the leading DNA strand by the DNA polymerase, resulting in chain termination, thus leading to a selective ablation of the CAR T cells [176].
- (ii) MAbs-based switches: A suicide switch approach for targeted ablation of CAR T cells involves the genetic modification of these cells to express a recombinant cell surface protein and the CAR simultaneously. This surface protein should possess a binding epitope recognizable by an established monoclonal antibody (such as EGFR-specific cetuximab or CD20-specific rituximab) in its conformationally intact form. This makes the CAR T cells susceptible to ADCC or CDC when exposed to the appropriate monoclonal antibody [177,178,179]. However, the clinical application of this approach using monoclonal antibodies as switches may be limited due to potential harm to healthy tissues expressing the native form of the recombinant protein upon administration of the monoclonal antibody (e.g., B-cell ablation upon Rituximab treatment).
- (iii) The inducible caspase (iCasp) switches involve the fusion of a modified human FK506-binding protein (FKBP12) to either the human caspase 9 or the membrane-anchored intracellular domain of Fas. Upon the introduction of a dimerizing drug, the modified FKBP12 binds to this drug with high affinity, enabling the dimerization and subsequent activation of the inducible caspase 9 (iCasp9) [175] or the Fas-based suicide switch [180]. This activation triggers downstream apoptotic cascades that result in the targeted ablation of CAR T cells carrying this switch.

As mentioned earlier, CAR T cells can be generated either by lentiviral transduction, leading to a stable expression of the CAR, or by electroporation, resulting in limited persistence of the CAR construct. Therefore, transfection of the CAR T cells with the CAR construct via nucleofection presents another approach to reducing on-target off-tumor toxicities [181]. However, a reduced efficacy has to be discussed upon nucleofection, leading to the need for sequential CAR T cell administrations.

### 4.5. Genetic Manipulation of CAR T Cells to Overcome Fratricide

The expression of the CAR target antigen on the surface of CAR T cells triggers fratricide, leading to compromised CAR T cell persistence and diminished antitumor efficacy of the adoptive cell therapy. Recent advances in genetic engineering have brought up techniques, that can be employed to avoid fratricide:

- (i) CRISPR/Cas9-based knockout of the target antigen: as already shown for CD7-targeting CAR T cells in *in vitro* and *in vivo* models of T-ALL [158], the knockout of the target antigen, while also transducing the same cell with a CAR construct for this antigen, will result in efficient targeting and killing of malignant T cells without significant effector T cell fratricide.
- (ii) Zinc finger nuclease (ZFN)-mediated loss of functional target gene expression: ZFNs function as genome editors by catalyzing DNA double-strand breaks (DSBs) in the genome, leading to permanent loss of functional target gene expression via repair by nonhomologous end joining [182]. Exemplarily, the application of ZFNs has been utilized to eliminate the expression of the α and β chains of the endogenous TCR in allogeneic T cells in a CD19-redirected CAR T cell product, leading to CAR T cells which did not respond to TCR stimulation [183]. ZFN-based gene editing has so far been not applied to T cell-directed CAR T cells.
- (iii) Transcription activator-like effector nuclease (TALEN)-assisted disruption of the endogenous target gene expression. TALENs are restriction enzymes that can be customized to cleave precise sequences of DNA [184]. In CD3-targeting CAR T cells, TALENs have been utilized to disrupt the endogenous TCR signaling cascade [185].

### 4.6. Generating Allogeneic CAR T Cells to Overcome Product Contamination

Generating CAR T cells from patients with T cell malignancies presents a challenge due to the difficulty of selecting only non-malignant T cells during the T cell isolation process. Malignant T cells could also be isolated alongside normal ones, as recently happened during the production of CD19 CAR T cells [186]. In order to prevent product contamination, the possibility of generating allogeneic CAR-T cells from a healthy third-party donor could be considered as a reasonable option. In contrast to autologous CAR T cells, allogeneic CAR T cells have a shorter *in vivo* persistence after infusion into patients. Additionally, the recipient’s immune system may rapidly attack and eliminate them. On the other hand, allogeneic CAR T cells can also induce life-threatening GvHD in the recipient [187]. Notably, the feasibility of allogeneic T cell-directed CAR T cell therapy has been proven for T-ALL (e.g., CD7-directed CAR T cells [158]). Therefore, the generation of allogeneic, off-the-shelf CAR T cells is one of the most promising approaches in the immunotherapy of MTCL/L.

To tackle the aforementioned constraints, researchers have devised allogeneic CAR T cell therapies that employ different techniques, such as the before-mentioned gene-editing approaches or suicide switches. In addition, instead of applying αβ T cells, γδ T cells present as a suitable platform for CAR-based, allogeneic adoptive cell therapy, as γδ T cells (i) have the capability to be expanded to larger numbers *ex vivo* [188], (ii) are considered to be incapable of mediating GvHD (as their TCR activation is not MHC-dependent) [189], and (iii) have the property of going into the intestine, skin, and reproductive system [190]. Notably, the application of unmodified γδT cells was already tested in the treatment of one T-NHL patient, utilizing a rather exotic strategy: the patient received *ex vivo* expanded allogeneic γδT cells obtained from a haploidentical donor, without any artificial alterations (such as CAR implementations) [191]. Nevertheless, he went into CR for 5 months without any signs of GvHD development. A Phase I clinical trial for r/r NHL, including PTCL, is currently ongoing (NCT04696705). Thus, utilizing donor-derived γδ T cells might serve as promising immunotherapy, either without any modifications or after the implementation of a CAR.

### 4.7. Utilizing Effector Cells other Than T Cells for CAR-Based Immunotherapy

Besides the usage of allogeneic T cells for CAR-based therapy of MTCL/L, utilization of other effector cells has emerged in the past, in order to prevent fratricide. In one approach, the natural killer (NK) cell line NK-92 has been utilized to engineer anti-CD3 CAR NK cells [170]. As NK cells do not present an expression of CD3, the limitation of fratricide can be overcome. The CD3 targeting CAR NK-cells showed promising selectivity as well as activity in various primary PTCL samples as well as MTCL/L cell lines *ex vivo* [170]. In addition, these cells effectively controlled and suppressed Jurkat tumor cell growth in *in vivo* xenograft models, resulting in a significant extension of mice survival times [170]. Applying the same technique, NK cells targeting CD5 have been developed, utilizing the NK-92 cell line. Using the same *ex vivo* as well as *in vivo* systems, which have been applied for the anti-CD3 CAR NK cells, a high anti-tumor activity has been demonstrated for anti-CD5 NK cells [172]. Notably, as NK-cells do not express a TCR, there is no risk for GvHD mediated by the CAR NK-cells [192]. However, it is important to state that CAR-expressing NK cells are not as long-lasting as conventional CAR T cells. In addition, the *in vitro* expansion as well as the introduction of the CAR construct is more challenging in comparison to CAR T cells [193]. Finally, the side effects of NK-cell infusions rarely seem to be manageable [194].

In addition to T cell-directed CAR NK-cell therapy, CAR-engineered macrophages (and other cells of the myeloid lineage) are on the horizon. In the treatment of r/r MTCL/L, a CD5-targeting CAR product, made with myeloid cells collected from the patient’s blood, is currently under investigation within a Phase I/II clinical trial (NCT05138458).

Summarizing, the foundation for CAR T cell therapies in MTCL/L has been laid: suitable special forces are T cells, γδ T cells, NK cells, and macrophages, which are armed with the CAR constructs and, if necessary, suicide switches or genetic disruption of the target antigen. Now, the right composition (selection of the right special force with suitable armament and promising target) has to be preclinically and especially clinically tested and its side effects have to be identified. Currently, most trials focus on CD30-directed CAR T cell therapies, often as a subcohort alongside Hodgkin lymphoma, but other CAR-based products additionally emerge for the treatment of MTCL/L.

## 5. Discussion

Clinical management of MTCL/L is especially challenging, as those mostly aggressive T cell malignancies are rare, resulting in a lack of easily executed clinical trials on novel substances. Furthermore, based on T cell-specific cellular characteristics, pathobiological mechanisms are complex. However, distinct MTCL/L were identified to share genomic hallmarks and make use of the same pathways for their oncogenic drive [195], allowing the overarching development of targeted treatment approaches directed against tumor-specific molecular mechanisms; for example, genomic aberrations of members of the Janus(JAK)/signal transducer and activator of transcription (STAT) signaling, leading to constitutive activation of this pathway [196]. In addition, alterations of epigenetic regulators (e.g., of histone deacetylases (HDAC)) and members of the phosphatidylinositol 3-kinases (PI3K)/protein kinase B (AKT) pathway are highly recurrent in MTCL/L [195]. Although targeted approaches inhibiting, e.g., JAK or mechanistic Target of Rapamycin (mTOR) show encouraging results in small case series, they did not result in a new era in the treatment of T cell entities [197]. Thus far, HDAC inhibition using Romidepsin showed promising results for the treatment of r/r PTCL [198]; this, however, could not be translated into treatment as a first-line therapy, as combinations of Romidepsin with CHOP were found to be inappropriate for improving progression-free survival of previously untreated PTCL patients [199,200].

**Figure 2:**
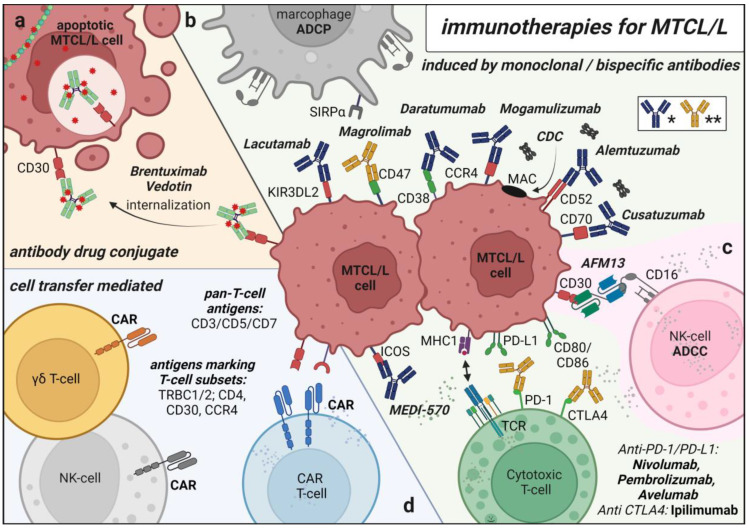
Schematical Overview of Immunotherapies for MTCL/L. (**a**) antibody drug conjugate Brentuximab–Vedotin for targeted chemotherapy delivery (**b**) monoclonal antibodies targeting MTCL/L cells for antibody dependent cell mediated phagocytosis (ADCP), antibody dependent cell mediated cytotoxicity (ADCC), complement dependent cytotoxicity (CDC) and monoclonal antibodies reactivating inhibited healthy T cells/macrophages (* blue: CDC/ADCC inducing antibody ** yellow: pathway inhibitory antibody) (**c**) bispecific NK-cell recruiting antibody AFM13 (**d**) cell transfer mediated therapies of autologous/allogenic T/NK cells genetically modified to express Chimeric Antigen receptors (CARs). Created with BioRender.com.

For immunotherapies, the molecular mechanisms of transformation into and maintenance of the malignant state are largely irrelevant (see exceptions for, e.g., CD38 targeting by Daratumumab). As especially ADCs, plain mAbs, and CAR-directed cellular therapies essentially depend on the surface expression of target structures, an MTCL/L subtype-specific response pattern is not to be expected (only if subtypes are defined by distinct T- or NK-cell immunophenotypes determining surface marker expression). Thus, future clinical efforts can benefit from testing novel therapeutic strategies in bigger patient cohorts that are heterogeneous with respect to disease entities, but homogeneous with respect to leukemia/lymphoma cell expression of targeted surface markers. In this review, we presented and discussed preclinical as well as clinical efforts, how special forces of the patient’s immune system can be educated, armed, and empowered to eradicate secretly defected (=malignant) T cells. We gave an overview of the various platforms of cancer immunotherapies available/in the pipeline for the treatment of MTCL/L, including monoclonal antibodies and cell-mediated treatment strategies. Presented approaches include both already established and approved immunotherapies (e.g., Alemtuzumab for T-PLL, BV for ALCL) and also currently tested immunotherapeutic approaches (e.g., CAR-T cells targeting malignant T cells) (see Figure 2 for schematic overview). Having seen the impressive therapeutic successes of mAbs-mediated cytotoxicities [33] and CAR T cell therapies in aggressive B-cell lymphoma [146,147] investigators are now challenged by the translation of those into the T- and NK-cell leukemia/lymphoma field. Achievements in the field of genome editing as well as the development of suicide switches (originally for the management of GvHDs) provide the toolbox for equipping CAR T cells with characteristics to overcome the challenges of fraternal death and product contamination, as well as T cell depletion. In addition, other distinct TSAs are currently in the pipeline that will likewise enable improved targeted mAb mediation as well as CAR T cell therapies. The ultimate goal should be to develop, e.g., an off-the-shelf (and thus allogeneic) CAR T cell product that either targets a defined TSA or is genetically modified to not express the target gene itself. The first case series in T-ALL are encouraging and show what success is possible in relapsed/refractory T-NHL [201]. To achieve this, joint efforts, culminating in a multicenter study, are highly warranted to ensure sufficient case numbers leading to a meaningful study that also detects rare side effects. Apart from CAR T cell therapies, strategies involving patients’ innate immune cells seem especially promising, with, e.g., the ‘off-shelf-available’ NK-cell recruiter (bispecific antibody) AFM13 for PTCL/CTCL. Future approaches might take advantage of combining immunotherapies with small molecules targeting oncogenic pathways deregulated in PTCL.

## 6. Conclusions

Based on the presented studies, we conclude that immune system-based treatment strategies for targeting MTCL/L T cells are overall very promising with excellent chances for success, depending on the targeted entity and the chosen treatment strategy. Overall, two lines of attack have to be followed: (i) if taking advantage of the patient’s immune system, further research should focus on physiological processes afflicted with the innate immune system rather than T cell mediated immunity (refrain from following, e.g., targeting the PD-1/PD-L1 immune-checkpoint and focus on, e.g., bispecific antibodies such as AFM13, that can recruit NK cells towards target antigen carrying lymphoma cells) and (ii) if utilizing CAR-T-cell therapies, make use of intelligent genetical engineering to overcome specific hurdles when targeting T cells using T cells.
